# Inequity in colorectal cancer treatment and outcomes: a population-based study

**DOI:** 10.1038/sj.bjc.6604467

**Published:** 2008-07-01

**Authors:** A-E Carsin, L Sharp, D P Cronin-Fenton, A Ó Céilleachair, H Comber

**Affiliations:** 1 National Cancer Registry, Ireland, Building 6800, Cork Airport Business Park, Kinsale Road, Cork, Ireland; 2 Department of Clinical Epidemiology, Aarhus University Hospital, Ole Worms Alle 1150, Aarhus C 8000, Denmark

**Keywords:** colorectal cancer, resection, chemotherapy, radiotherapy, survival

## Abstract

Several uncertainties surround optimal management of colorectal cancer. We investigated treatment patterns and factors influencing treatment receipt and mortality in routine clinical practice. We included 15 249 individuals, recorded by the National Cancer Registry (Ireland), with primary invasive colon or rectal tumours, diagnosed during 1994–2002. Logistic regression and Cox proportional hazards were used to determine factors associated with treatment receipt within 1 year of diagnosis and with mortality, respectively. A total of 78% had colorectal resection, 31% chemotherapy, and 13% radiotherapy (4% colon; 28% rectum). Half of stage IV patients underwent resection. Chemotherapy and radiotherapy use increased by at least 10% per annum. There was a notable increase in pre-operative radiotherapy from 2000 onwards. Patient-related factors were significantly associated with treatment receipt. Patients who were male, older, not married, or smokers had significantly higher risks of death. Chemotherapy was significantly associated with lower mortality for stage III, but not stage II, colon cancer. For rectal cancer, pre-operative radiotherapy was associated with reduced mortality. Surgery and chemotherapy were associated with longer survival for stage IV patients. The observed inequities in treatment and outcomes suggest that there is potential for further dissemination of therapies in routine practice. Improving treatment availability overall, and equity, has the potential to reduce mortality.

Colorectal cancer is responsible for more than half a million deaths worldwide each year (Ferlay *et al*, 2004). In many developed countries, mortality rates have been falling for 20–30 years (Coleman *et al*, 1993), reflecting steady improvements in survival (Verdecchia *et al*, 2007). However, there remains considerable international variation in survival even between developed nations; 5-year relative survival for patients in the USA is around 65%, whereas that within Europe ranges between 45 and 64% (Verdecchia *et al*, 2007). Differences in stage at diagnosis, although important (Gatta *et al*, 2000; Ciccolallo *et al*, 2005), do not completely explain survival inequalities. Variations in treatment use and quality may also play a role.

Several uncertainties surround optimal colorectal cancer therapy, and this is reflected in variations in best-practice guidelines. Although surgery is the cornerstone of treatment for apparently localized disease, international variations have been reported in resection rates (Ciccolallo *et al*, 2005). For locally advanced or metastatic disease, there is no consensus on when excision of the primary tumour should be attempted (SIGN, 2003; NCCN, 2006a, 2006b). Use of adjuvant therapy depends both on site and stage. For example, adjuvant chemotherapy is not routinely recommended for stage II colon cancer (SIGN, 2003; Australian Cancer Network Colorectal Cancer Guidelines Revision Committee, 2005) given the inconclusive findings from randomised controlled trials (RCTs) (Mamounas *et al*, 1999; Benson *et al*, 2004). However, some recent studies suggest that stage II patients with poor prognostic features (such as bowel perforation, involved lymph nodes, etc.) might benefit (Gill *et al*, 2004; André *et al*, 2006) and US and Australian guidelines advocate chemotherapy for this subgroup (Australian Cancer Network Colorectal Cancer Guidelines Revision Committee, 2005; NCCN, 2006a). For stage III colon cancer, RCTs suggest that best practice is surgery followed by chemotherapy (5-fluorouracil (5-FU)–leucovorin or capecitabine, and more recently supplementation with oxaliplatin) to improve survival (Cascinu *et al*, 2003; André *et al*, 2004a; Twelves *et al*, 2005). In advanced disease, irinotecan or oxaliplatin with 5-FU is recommended for primary therapy (NICE, 2005). For rectal cancer, until recently post-operative radiotherapy was the standard of care for locally advanced disease, but RCTs have indicated that pre-operative administration may improve local control, with lower toxicity and reduced rectal cancer deaths (Colorectal Cancer Collaborative Group, 2001; Sauer *et al*, 2004). The addition of chemotherapy improves response and is recommended (SIGN, 2003; Australian Cancer Network Colorectal Cancer Guidelines Revision Committee, 2005).

How these uncertainties and complexities translate into routine clinical practice is not clear. Some studies have suggested that (neo-) adjuvant therapies may be under-utilized and that use depends on patient characteristics such as age or ethnicity (Schrag *et al*, 2001a, 2001b; Potosky *et al*, 2002; Martijn *et al*, 2003; Cronin *et al*, 2006). However, most population-based studies originate from the United States, relate to patients diagnosed in the first half of the 1990s, and/or focus on patient subgroups (e.g. stage III disease). We therefore undertook a population-based investigation of trends in treatment and factors predicting treatment receipt, survival, and mortality for patients with all stages of disease during 1994–2002 in Ireland – a country with relatively high colorectal cancer mortality (Ferlay *et al*, 2004).

## Materials and Methods

Details of individuals aged ⩾20 with invasive colorectal cancer (ICD-02: C18-20) diagnosed during 1994–2002 were abstracted from the National Cancer Registry (NCR), which records all cancers diagnosed in Ireland (www.ncri.ie). Completeness of registration is approximately 98% (NCR, 2001). Cases were excluded where diagnosis was made by death certificate only or at autopsy, or the tumour was a secondary malignancy (other than non-melanoma skin), or occurred simultaneously with another tumour.

Using information on treatments administered within 1 year of diagnosis, cases were categorised by receipt of (1) ‘cancer-directed’ surgery (i.e. colon or rectal resection), (2) chemotherapy, or (3) radiotherapy. Chemotherapy and radiotherapy were classified as ‘any’, ‘pre-operative’, or ‘post-operative’ based on dates of surgery and the start of the first treatment course. Stage was defined by AJCC summary staging (AJCC, 2002), making the assumption that patients with no information on distant metastasis (MX) had no metastasis (M0). Dates and causes of death were ascertained by linkage to death certificates. Patients were followed up from date of diagnosis to death or 31 December 2004, whichever was sooner.

Associations between patient characteristics and stage were evaluated using *χ*^2^ tests. Temporal trends in treatment were explored by jointpoint regression (Edwards *et al*, 2005). Multivariate logistic regression models, stratified by stage, were built to identify factors associated with treatment receipt. For surgery, only stage IV and unstaged patients were modelled, as almost all stage I–III patients had resection. For chemotherapy and radiotherapy, stage I patients were excluded as few received these therapies. Models were adjusted for sex and factors significant (*P*<0.10) on likelihood ratio tests. Factors considered for inclusion were age, marital status, site, grade, tumour extent (T), regional lymph node status (N) (as within a given stage, subgroups of patients might receive adjuvant therapy), year of diagnosis, smoking status at diagnosis, and health board of residence (as cancer services were organised at this level in 1994–2002). Model goodness of fit was assessed by the Hosmer–Lemeshow test. Multivariate Cox proportional hazard models were run for all-cause and colorectal cancer-specific mortality. As results were similar, only all-cause mortality is presented. Surgical and non-surgical patients were modelled separately and covariates included if significant (*P*<0.10) on Wald F-tests. Owing to non-proportional hazards, chemotherapy and radiotherapy were included as time-varying covariates with date of the start of the first course as the index date. In all models we tested for interactions between variables; any that were meaningful, statistically significant, and impacted on risk estimates were included in the final models. Kaplan–Meier curves, adjusted for age, were constructed to illustrate the impact of treatment on survival time.

## Results

Colon cancer accounted for 63% of the 15 249 patients included. Overall, 57% were male (53% of colon, 64% of rectal tumours) and 92% had histologically verified disease. Less than 12% were stage I, 25% were stage II, 23% were stage III, 22% were stage IV, and 17% were unknown stage. Unstaged patients were older and more often had rectal tumours. Stage distribution changed relatively little over time (stage I/II: 39% in 1994–96 and 36% in 2000–02; stage III/IV: 43 and 48%; unstaged: 18 and 15%).

### Treatment rates and trends over time

Overall, 78% of patients had surgical resection, 31% chemotherapy, and 13% radiotherapy, the latter primarily for rectal cancer (28% rectal, 4% colon; Table 1). Almost all stage I–III patients (96%) had surgery, compared with 51% with stage IV and 47% with unknown stage. Chemotherapy and radiotherapy were used almost exclusively among surgical patients. For rectal cancer, both therapies were more often used post-operatively (chemotherapy 31%, radiotherapy 16%) than pre-operatively (6 and 8%, respectively). Overall, 17% of patients received no cancer-directed treatment; almost all of these had stage IV (45%) or unstaged (47%) disease.

**Figure 1:**
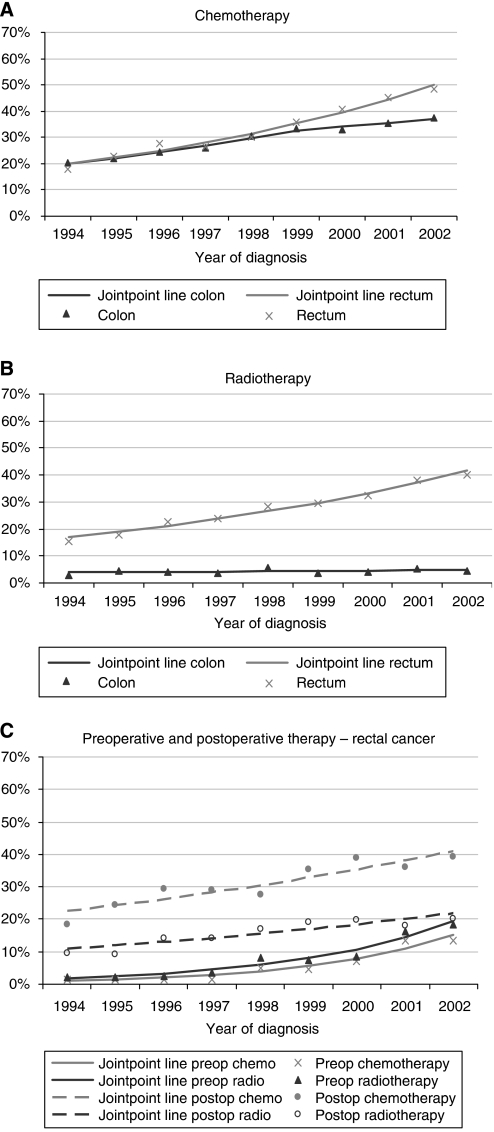
Trends in treatment receipt among colorectal cancer patients, 1994–2002: observed frequencies plus jointpoint regression lines. (**A**) chemotherapy (% of all patients), (**B**) radiotherapy (% of all patients), and (**C**) pre and postoperative therapy (% of rectal cancers).

From 1994 to 2002, there was no change in the proportion undergoing surgery (data not shown). Chemotherapy use increased significantly for both colon and rectal cancer (estimated annual percentage change (EAPC)=+9.6%, 95% CI +8.4, +10.8%; Figure 1A), although the increase slowed down for colon cancer patients in 2000–02. The growth in chemotherapy was evident for all disease stages. Use of radiotherapy for rectal cancer increased overall (EAPC=+12.3%, 95% CI +10.3, +14.2%; Figure 1B), particularly for those with stage I disease (EAPC=+23.0%, 95% CI +16.0, +30.3%). The upward trend was most pronounced for pre-operative use (EAPC=+34.3%, 95% CI +24.7, +44.6%; Figure 1C), particularly from 2000 onwards.

### Factors associated with treatment receipt

With regard to surgery for stage IV disease, in multivariate analyses, resection was significantly less common among patients who were older, unmarried, and male (*P*⩽0.01; data not shown) and there was a borderline significant effect of site (multivariate odds ratio (OR) rectum *vs* colon=0.87, 95% CI 0.75–1.01). For unknown stage disease, older, unmarried patients were significantly less likely to undergo resection (*P*<0.001; data not shown), as were those with tumour extent T4 (OR=0.07, 95% CI 0.05–0.11).

Table 2 shows factors associated with chemotherapy receipt. For all stages, older and unmarried patients were significantly less likely to receive chemotherapy. For stage III and IV patients, there was a strong positive effect of year of diagnosis. For stage II and unknown stage patients, there was year of diagnosis–site interaction; chemotherapy use increased over time, but to a greater extent for rectal than colon patients. Tumour extent was associated with likelihood of chemotherapy receipt. For stage II patients, those with T4 tumours were significantly more likely to be treated than those with T3 tumours (multivariate OR=1.69, 95% CI 1.33–2.15). For III, IV, and unstaged patients, those with T3 tumours were most likely to have chemotherapy.

Table 3 shows factors associated with radiotherapy use in rectal cancer. For all disease stages, use decreased significantly with increasing age. Women were slightly less likely to get radiotherapy than men, and this was significant for stage II disease. For stage III patients, use was less common among those who were not married (*P*=0.055). Tumour extent was significantly associated with radiotherapy receipt in patients with unknown and stage II disease, but not stage III and IV disease. Among those with stage II or unknown stage disease, patients with T4 tumours were more likely to receive radiotherapy than those with other tumours.

With regard to pre-operative radiotherapy specifically, use was significantly less frequent among female patients (multivariate OR=0.67, 95% CI 0.51–0.87) and decreased with age (*P*(trend)<0.001; data not shown).

### Survival and factors associated with mortality

Five-year observed survival was 40% overall, and 72, 59, 41, 7, and 31% for stage I, II, III, IV, and unstaged tumours, respectively. It was 49% for surgical and 9% for non-surgical patients. Among stage IV patients, 50% who underwent resection were alive at 1 year compared with 16% who did not.

Table 4 shows factors associated with mortality, stratified by surgery receipt. The risk of death fell over time for surgical patients. In both groups, men had significantly higher hazard ratios (HR) than women, and married patients had lower hazards (*P*⩽0.01). The HR increased with age, particularly for older surgical patients. Patients who presented with later stage disease and with poorly differentiated or undifferentiated tumours had significantly higher hazards (*P*⩽0.01). Smokers had increased hazards, and this reached significance among surgical patients.

**Figure 2:**
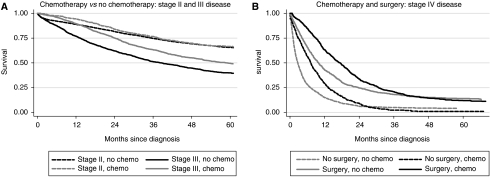
Kaplan–Meier survival curves (adjusted for age) by receipt of treatment: colorectal cancer.

Table 5 shows HRs for chemotherapy and radiotherapy receipt, stratified by stage, and Figure 2 shows survival according to treatment. Stage III patients who received chemotherapy had a significantly reduced risk of death (all patients HR=0.67, 95% CI 0.61–0.75) and higher survival (Figure 2A). No such effects were evident for stage II disease. For stage IV disease, surgical patients had higher survival; this was evident up to approximately 3 years after diagnosis among the group who had surgery and up to 2 years after diagnosis among those who did not have surgery (Figure 2B). Among surgical stage IV patients, chemotherapy receipt was associated with a significantly reduced risk of death (HR=0.77, 95% CI 0.68–0.88). For rectal cancer, receipt of any radiotherapy (pre- or post-operative) had no significant impact on mortality for patients with any stage of disease (Table 5). However, when the analysis was limited to patients who had surgery and radiotherapy, those who received pre-operative, as compared with post-operative, radiotherapy had reduced HRs. This was seen for all stages of disease except stage II, but the reduction in the HR reached statistical significance only for stage III disease (HR=0.61, 95% CI 0.42–0.91).

## Discussion

### Strengths and limitations

This is one of the largest population-based studies of treatment trends and factors predicting treatment receipt for colorectal cancer, and is one of the few studies conducted outside the USA. We have described treatment patterns in routine clinical practice, rather than in specialized treatment centres, and in a setting with relatively high colorectal cancer mortality (Ferlay *et al*, 2004). With regard to limitations, we did not have details of specific chemotherapy regimens or radiotherapy courses. However, assessment of such details was not our aim. Although we only had information on treatments received within a year of diagnosis, most active treatment for the primary tumour would be offered within this time. *A priori*, we could not distinguish whether treatments were given with curative or palliative intent. Stratification of the analysis by stage and investigation of treatment combinations helped clarify this to some extent.

For 40% of the cases information on T, N, and M status was incomplete. The registration procedures of the National Cancer Registry include medical record review, so these data would have been recorded had they been available in notes. In a substantial proportion of these cases, it is probable that the investigations to confirm presence – or more likely absence – of metastases were not carried out. We coded cases with missing metastasis data to no metastasis, reducing the proportion ‘unstaged’ to 17%. The validity of this assumption was verified by repeating the analyses after assigning patients with missing metastasis data to the unstaged group – our results were not altered.

Other than stage and grade, no prognostic information (e.g. bowel perforation, number of lymph nodes examined, etc.) was available. Such factors are likely to have determined treatment aggressiveness and survival, especially among stage II colon cancer patients, who comprise a particularly heterogeneous group (André *et al*, 2006).

### Treatment rates and trends over time

The colorectal resection rate was constant over time, in contrast to a US study that reported a slight, but significant, decrease during 1988–2000 (Cook *et al*, 2005). Our frequency of 78% undergoing resection was lower than that in US studies (90–92%; Ciccolallo *et al*, 2005) but close to figures for European community practice (Gatta *et al*, 2000; Pitchforth *et al*, 2002). The differences may be partly a result of variations in stage distribution between populations, but are more likely to be due to lack of consensus regarding resection of the primary tumour in stage IV patients (Rosen *et al*, 2000; Sarela *et al*, 2001; Ruo *et al*, 2003). In two US population-based studies, 66% of stage IV patients of all ages (Cook *et al*, 2005) and 72% of those aged ⩾65 had primary cancer-directed surgery (Temple *et al*, 2004); this was 51% in our study overall.

One of the major reasons for examining treatment patterns in routine clinical practice is to determine whether potential exists for further dissemination of therapies across the patient population. The increased use of chemotherapy and radiotherapy over time (10 and 12% per annum, respectively) suggests some dissemination of RCT-approved therapies into community practice. The dramatic increase in pre-operative radiotherapy for rectal cancer from 2000 onwards is similar to recent US trends (Cronin *et al*, 2006). It is noteworthy, however, that post-operative radiotherapy is still commonly used in our population, in contrast to the USA and the Netherlands, where there has been a shift to pre-operative administration (Martijn *et al*, 2003; Cronin *et al*, 2006). In the Netherlands this was due to implementation of new guidelines, and illustrates the potential for these (which do not exist in Ireland) to influence patient management in routine practice.

Use of chemotherapy was slightly lower in our population than in US community practice (Jessup *et al*, 2005; Cronin *et al*, 2006). This was particularly true for older patients: 38% of stage III patients over 65 in Ireland had chemotherapy compared with 55% in the United States in 1991–96 (Schrag *et al*, 2001a). Similarly, only 24% of over 65 with stage IV disease had chemotherapy compared with 44% in the United States in 1991–99 (Temple *et al*, 2004). Somewhat more reassuringly, when compared with other European population-based series from the 1990s, utilization of chemotherapy in our population did not appear to be unusually low (Bouchardy *et al*, 2001; Faivre-Finn *et al*, 2002). For rectal cancer, however, radiotherapy use was low compared to both US and European populations (28% in Ireland *vs* 46–62% elsewhere) (Ayanian *et al*, 2003; Cronin *et al*, 2006; Vulto *et al*, 2007). Possible reasons for this include a lack of guidelines, lack of centralisation and specialisation of cancer services during the study period, and a shortfall in radiation oncology services (Expert Working Group on Radiation Oncology Services, 2003).

In our study, 17% of patients did not receive any cancer-directed treatment. This group comprised mainly elderly patients who presented late and for whom treatment options were likely to be limited. Almost 60% were 75 or older at diagnosis and a further 27% were 65–74 years; 45% were stage IV and 47% had unknown stage disease. The median survival of the entire group was only 2 months. Whether this might have been longer had some of these individuals received treatment (e.g. by irinotecan or oxaliplatin with 5-FU; NICE, 2005) is open to speculation.

### Factors associated with treatment

As only 4% of stage I–III cases did not have surgery, we were unable to investigate factors associated with not undergoing resection for these patients. For stage IV disease, the likelihood of colorectal resection declined with increasing age. This was also observed in two US series (Temple *et al*, 2004; Cook *et al*, 2005), although in both series the effect was mainly limited to the very old (80 or 85 and over), whereas in our study there was a steady decrease with increasing age (60% of the under 65s had a resection, whereas only 51% aged 65–74 and 42% aged ⩾75 did). In addition to having more co-morbid conditions (Yancik *et al*, 1998), older patients are more likely to require emergency surgery (Colorectal Cancer Collaborative Group, 2000a), and these factors may influence the proportion resected in different populations. We also found that female, stage IV patients were significantly more likely to undergo resection, which has not been seen elsewhere. The reason for this is unclear, but co-morbidities may be relevant here also; Yancik *et al* (1998) found that female patients were less likely than male patients to have a high-impact life-threatening co-morbid condition.

With regard to adjuvant treatment, we observed significant disparities in use even after adjusting for stage and other clinical factors. The increased likelihood of treatment in married patients was also noted in a small chemotherapy study (Bouchardy *et al*, 2001). The explanation is not clear but possibilities include active involvement of spouse/family members in care management, spousal support (emotional and logistical) during treatment, and different perceptions – by clinicians or patients – of the ‘value’ of life in those with and without a spouse and/or dependants. Age was the strongest predictor of receipt of chemotherapy or radiotherapy. Although reported elsewhere (Faivre-Finn *et al*, 2000; Schrag *et al*, 2001a, 2001b; Cronin *et al*, 2006), this finding remains important given that one-third of colorectal cancer patients are 75 or older. Although the higher prevalence of co-morbidities (Yancik *et al*, 1998) may preclude treatment in some elderly patients, in the United States the age effect persists even among those without co-morbidities (Schrag *et al*, 2001a, 2001b). Older patients may also have increased treatment-related toxicity, although evidence on this is mixed (Sargent *et al*, 2001). Both RCTs (Sargent *et al*, 2001) and observational studies (Bouchardy *et al*, 2001; Schrag *et al*, 2001a; Jessup *et al*, 2005) demonstrate clear survival benefits of adjuvant chemotherapy in patients of all ages, suggesting that there may be further potential for (and benefits to be gained from) extended use in older patients in routine practice.

Randomised controlled trials suggest that chemotherapy may benefit stage II colon patients with poor prognostic features (Gill *et al*, 2004; Australian Cancer Network Colorectal Cancer Guidelines Revision Committee, 2005; André *et al*, 2006). These findings appear to have disseminated into routine practice, as those stage II patients in our study with more extensive tumours were significantly more likely to be treated.

### Factors associated with mortality

Among surgical patients, risks of death decreased over time, which may be partly due to reductions in post-operative mortality (Faivre-Finn *et al*, 2002). The increased risk of death with increasing age among surgical patients may also be a function of post-operative mortality. In further analyses, patients aged ⩾75 were four times more likely to die in the 30-day post-operative period than younger patients (HR=4.03, 95% CI 2.46–6.59).

The increased hazard in those who were smokers at diagnosis is intriguing. As smoking status is based on information recorded in medical records, misclassification is probable, but it is most likely that some smokers would have been categorized as non-smokers rather than the other way; thus, the observed result probably underestimates the true effect. The finding does not seem to be due to deaths from other causes in smokers, as the association persisted when cause-specific mortality was analysed. Two small studies have reported poorer outcomes in smokers following colorectal cancer surgery (Jadallah *et al*, 1999; Munro *et al*, 2006). Although the result could be due to confounding by another prognostic factor such as deprivation (Bouchardy *et al*, 2001; Pitchforth *et al*, 2002; Wrigley *et al*, 2003), there are several potential mechanisms by which smoking might adversely affect survival. These include effects on immune function (O’Byrne *et al*, 2000), inflammatory response (Yanbaeva *et al*, 2007), metabolism of chemotherapy drugs (van der Bol *et al*, 2007), and genetic damage and repair capacity (Fracasso *et al*, 2006).

### Treatment, survival, and mortality

We investigated the impact of treatment on mortality and survival primarily to determine whether the advances seen in RCTs have translated into improvements for the entire patient population. Clearly, survival comparisons in an observational study are potentially subject to bias and will tend to favour treatment (because of the selection of patients with better prognostic features for treatment). Although the introduction of time-varying covariates, and adjustment for other factors, would be expected to attenuate the bias somewhat, we cannot exclude the possibility that our results are influenced by unmeasured confounders. The Kaplan–Meier survival curves may be particularly susceptible in this regard, as they are adjusted for age only.

Given the controversy around resection of the primary tumour in stage IV patients, our observations of lower mortality and longer survival among resected patients at the population-level are noteworthy. In particular, the survival advantage conferred by the combination of chemotherapy and surgery is important and compatible with RCT evidence on prolonged survival for advanced patients treated with palliative chemotherapy (Colorectal Cancer Collaborative Group, 2000b). Similar results have recently been reported in the US population-based studies (Temple *et al*, 2004; Cook *et al*, 2005) and clinical series (Ruo *et al*, 2003; Konyalian *et al*, 2007). Given that stage IV disease is not uncommon (it accounted for more than one-fifth of our cases), the role of resection of the primary tumour (in combination with chemotherapy) warrants further investigation. Issues such as impact of treatment on quality of life need to be addressed.

The higher survival and significant mortality reduction for stage III patients receiving chemotherapy at the population level is consistent with evidence of survival benefits for colon cancer from RCTs (André *et al*, 2004a). The lack of a survival advantage for chemotherapy among stage II patients overall is also compatible with RCT evidence which suggests that the survival improvement, if any, is small (Benson *et al*, 2004; André and de Gramont, 2004b). As we were not able to clearly distinguish stage II patients with particularly poor prognostic features, we cannot exclude a beneficial effect of treatment at the population level in this subgroup.

Although post-operative radiotherapy has been relatively long established as the standard of care for rectal cancer, we found no clear benefit in terms of survival or mortality at the population level in stage II–III patients. This is consistent with a systematic review of 22 RCTs, which concluded that the impact on survival was marginal (Colorectal Cancer Collaborative Group, 2001). In the current study, risk of death was decreased when radiotherapy was administered pre-operatively although the findings were based on small numbers and were not statistically significant for stage II disease. Although RCTs suggest that pre-operative radiotherapy reduces rectal cancer deaths, this appears to be at the cost of increased risk of death from other causes (Colorectal Cancer Collaborative Group, 2001). Our results, based on deaths from all causes, suggest that advantages may outweigh disadvantages at the population level, at least for patients diagnosed with stage III disease.

As has been observed elsewhere (Verdecchia *et al*, 2007), colorectal cancer survival in Ireland is rising (Walsh and Comber, 2007). As the proportion of early-stage tumours did not increase over time and no screening is in place, it is unlikely that this improvement is due to earlier detection. Instead it may reflect the increased use of (neo-) adjuvant treatment that we have shown here – as well as other treatment-related factors such as advancements in surgical techniques, increased specialization, and so on. Despite the improvement, survival in Ireland remains below US figures and the EU average (Verdecchia *et al*, 2007). Although these differences are probably due in part to stage (Gatta *et al*, 2000), the relatively lower rates of cancer-directed treatment that we describe probably play a role.

## Conclusions

While recognizing that not all patients are suitable candidates for surgery or (neo-)adjuvant therapy, our findings suggest that there is potential for extended dissemination of therapies in routine clinical practice – both overall and in particular patient subgroups. Improving treatment availability generally, and equity specifically, has the potential to increase survival and ultimately reduce mortality at the population level.

## Figures and Tables

**Table 1:** Treatments administered to colorectal cancer patients diagnosed during 1994–2002: numbers and percentages of all patients

	**Colon**		**Rectum**		**Total**	
**Variable**	**No.**	**%**	**No.**	**%**	**No.**	**%**
*Overall treatment* a						
Surgery^b^	7591	79.0	4285	76.0	11 876	77.9
Any chemotherapy	2807	29.2	1873	33.2	4680	30.7
Any radiotherapy	410	4.3	1578	28.0	1988	13.0
						
Pre-operative treatment^c^						
Chemotherapy	45	0.6	240	5.6	285	2.4
Radiotherapy	11	0.1	339	7.9	350	2.9
						
Postoperative treatment^c^						
Chemotherapy	2435	31.9	1336	31.0	3771	31.6
Radiotherapy	227	3.0	682	15.8	909	7.6
						
*Treatment combinations*						
Surgery only^b^	5000	52.0	2419	42.9	7419	48.7
Chemotherapy only	304	3.2	123	2.2	427	2.8
Radiotherapy only	41	0.4	174	3.1	215	1.4
Surgery and radiotherapy	116	1.2	302	5.4	418	2.7
Surgery and chemotherapy	2250	23.4	648	11.5	2898	19.0
Chemotherapy and radiotherapy	28	0.3	186	3.3	214	1.4
Surgery, radiotherapy, and chemotherapy	225	2.3	916	16.3	1141	7.5
No cancer-directed treatment	1649	17.2	868	15.4	2517	16.5

a Categories not mutually exclusive; figures do not sum to 100%.

b Resection of the colon or rectum.

c Percentage of surgical patients.

**Table 2:** Factors associated with chemotherapy receipt among colorectal cancer patients diagnosed during 1994–2002: observed and adjusted percentages, multivariate ORs^a^ with 95% CIs, and *P*-values

	**Stage II (*n*=3879)**			**Stage III (*n*=3553)**			**Stage IV (*n*=3308)**			**Stage unknown (*n*=2620)**		
	**obs%**	**adj%**	**OR (95% CI)**	**obs%**	**adj%**	**OR (95% CI)**	**obs%**	**adj%**	**OR (95% CI)**	**obs%**	**adj%**	**OR (95% CI)**
*Sex*												
Male	30	22	1 (ref)	54	50	1 (ref)	40	32	1 (ref)	14	6	1 (ref)
Female	26	21	0.92 (0.78, 1.08)	49	52	1.06 (0.90, 1.25)	34	31	0.95 (0.79, 1.13)	11	6	0.96 (0.72, 1.26)
			*P*=0.308			*P*=0.478			*P*=0.563			*P*=0.750
*Age*												
<55	62	61	1 (ref)	79	80	1 (ref)	68	66	1 (ref)	36	29	1 (ref)
55–64	48	46	0.56 (0.44, 0.72)	72	72	0.65 (0.49, 0.85)	57	53	0.60 (0.46, 0.77)	26	19	0.59 (0.40, 0.87)
65–74	28	27	0.24 (0.19, 0.30)	55	56	0.32 (0.25, 0.42)	37	34	0.27 (0.21, 0.34)	15	10	0.27 (0.18, 0.40)
75+	6	5	0.04 (0.03, 0.05)	19	18	0.05 (0.04, 0.07)	10	10	0.06 (0.04, 0.08)	3	2	0.05 (0.03, 0.08)
			*P*<0.001			*P*<0.001			*P*<0.001			*P*<0.001
*Marital status*												
Married	35	24	1 (ref)	62	57	1 (ref)	47	37	1 (ref)	18	7	1 (ref)
Not married/ unknown	19	18	0.69 (0.58, 0.82)	38	43	0.55 (0.47, 0.65)	26	25	0.58 (0.49, 0.70)	9	5	0.73 (0.55, 0.96)
			*P*<0.001			*P*<0.001			*P*<0.001			*P*=0.026
												
Year of diagnosis			—			1.26 (1.22, 1.30)			1.21 (1.17, 1.25)			—
						*P*<0.001			*P*<0.001			
*Site*												
Colon			—	49	49	1 (ref)	36	30	1 (ref)			—
Rectum			—	56	55	1.26 (1.07, 1.49)	41	34	1.16 (0.97, 1.39)			—
						*P*=0.006			*P*=0.100			
*Site × year* b												
Colon, 1994–96	20	14	1 (ref)			—			—	9	4	1 (ref)
Colon, 1997–99	29	23	1.87 (1.47, 2.38)			—			—	9	4	0.91 (0.56, 1.48)
Colon, 2000–02	29	25	2.08 (1.63, 2.67)			—			—	9	5	1.08 (0.65, 1.79)
Rectal, 1994–96	20	13	0.93 (0.66, 1.31)			—			—	9	4	1.02 (0.62, 1.70)
Rectal, 1997–99	34	26	2.22 (1.65, 2.99)			—			—	17	8	2.05 (1.31, 3.21)
Rectal, 2000–02	48	39	4.05 (3.01, 5.44)			—			—	25	16	4.14 (2.64, 6.48)
			*P*<0.001									*P*<0.001
*Tumour extent*												
T1			—	48	36	0.50 (0.25, 1.04)	18	16	0.34 (0.13, 0.84)	3	1	0.06 (0.03, 0.13)
T2			—	51	49	0.88 (0.68, 1.13)	39	35	1.00 (0.67, 1.49)	12	4	0.27 (0.16, 0.46)
T3	27	20	1 (ref)	52	53	1 (ref)	46	35	1 (ref)	27	15	1 (ref)
T4	36	30	1.69 (1.33, 2.15)	49	47	0.78 (0.63, 0.97)	40	31	0.84 (0.68, 1.04)	20	13	0.89 (0.59, 1.36)
Missing			—	31	29	0.37 (0.14, 0.93)	24	27	0.68 (0.51, 0.90)	9	5	0.33 (0.23, 0.47)
			*P*<0.001			*P*=0.017			*P*=0.020			*P*<0.001
*Nodes*												
N0			—			—	35	28	1 (ref)			—
N1/N2			—			—	52	40	1.68 (1.31, 2.15)			—
Missing			—			—	25	26	0.87 (0.65, 1.16)			—
									*P*<0.001			

CI=confidence interval; OR=odds ratio.

a Odds ratios adjusted for factors shown in relevant column, plus health board and grade.

b Interaction term for site and period of diagnosis.

**Table 3:** Factors associated with radiotherapy receipt among rectal cancer patients diagnosed during 1994–2002: observed and adjusted percentages, multivariate ORs^a^ with 95% CIs, and *P*-values

	**Stage II (*n*=1143)**			**Stage III (*n*=1297)**			**Stage IV (*n*=1106)**			**Stage unknown (*n*=1204)**		
	**obs%**	**adj%**	**OR (95% CI)**	**obs%**	**adj%**	**OR (95% CI)**	**obs%**	**adj%**	**OR (95% CI)**	**obs%**	**adj%**	**OR (95% CI)**
*Sex*												
Male	35	31	1 (ref)	45	43	1 (ref)	25	23	1 (ref)	26	19	1 (ref)
Female	27	24	0.72 (0.53, 0.97)	39	39	0.84 (0.65, 1.09)	20	20	0.84 (0.62, 1.16)	21	17	0.89 (0.65, 1.21)
			*P*=0.028			*P*=0.193			*P*=0.297			*P*=0.452
*Age*												
<55	48	46	1 (ref)	64	65	1 (ref)	33	32	1 (ref)	51	48	1 (ref)
55–64	42	41	0.81 (0.53, 1.25)	48	47	0.48 (0.33, 0.70)	27	26	0.77 (0.50, 1.18)	36	32	0.51 (0.31, 0.85)
65–74	36	35	0.62 (0.41, 0.94)	45	45	0.45 (0.31, 0.64)	24	24	0.66 (0.45, 0.97)	30	25	0.37 (0.23, 0.60)
75+	13	11	0.15 (0.09, 0.24)	22	21	0.14 (0.09, 0.21)	14	13	0.33 (0.21, 0.53)	11	9	0.11 (0.07, 0.18)
			*P*<0.001			*P*<0.001			*P*<0.001			*P*<0.001
*Marital status*												
Married			—	48	44	1 (ref)			—			—
Not married/ unknown			—	35	38	0.77 (0.60, 1.01)			—			—
						*P*=0.055						
*Tumour extent*												
T1			—	28	22	0.41 (0.16, 1.08)			—	7	4	0.09 (0.04, 0.20)
T2			—	40	39	0.94 (0.66, 1.33)			—	21	15	0.41 (0.22, 0.75)
T3	31	27	1 (ref)	43	41	1 (ref)			—	40	31	1 (ref)
T4	42	39	1.70 (1.14, 2.53)	48	48	1.35 (0.93, 1.96)			—	41	40	1.50 (0.88, 2.54)
Missing			—	47	54	1.73 (0.56, 5.33)			—	21	18	0.50 (0.33, 0.75)
			*P=*0.009			*P*=0.124						*P*<0.001
Year of diagnosis			1.21 (1.15, 1.28)			1.19 (1.13, 1.25)			1.14 (1.08, 1.21)			1.21 (1.13, 1.29)
			*P*<0.001			*P*<0.001			*P*<0.001			*P*<0.001

CI=confidence interval; OR=odds ratio.

a Odds ratios adjusted for factors shown in relevant columns. Stage II, III, and unknown stage disease are also adjusted for health board. Stage IV and unknown stage disease are also adjusted for grade.

**Table 4:** Factors associated with risk of death among colorectal cancer patients diagnosed during 1994–2002, stratified by receipt of surgery: HRs,^a^ 95% CIs, and *P*-values

	**No surgery^b^ (*n*=3373)**	**Surgery^b^ (*n*=11876)**
	**HR (95% CI)**	**HR (95% CI)**
*Sex*		
Male	1.00	1.00
Female	0.90 (0.84, 0.98)	0.81 (0.77, 0.85)
	*P=*0.010	*P*<0.001
*Age*		
<55	1.00	1.00
55–64	1.10 (0.94, 1.29)	1.12 (1.02, 1.23)
65–74	1.41 (1.22, 1.63)	1.42 (1.30, 1.55)
75+	1.70 (1.47, 1.96)	2.16 (1.97, 2.37)
	*P*<0.001	*P*<0.001
*Stage*		
I	0.48 (0.32, 0.71)	0.36 (0.33, 0.40)
II	0.76 (0.58, 0.98)	0.58 (0.54, 0.62)
III	1.00	1.00
IV	2.31 (1.91, 2.78)	2.83 (2.64, 3.03)
Unknown	1.33 (1.10, 1.61)	0.69 (0.63, 0.76)
	*P*<0.001	*P*<0.001
*Marital status*		
Married	1.00	1.00
Not married/unknown	1.12 (1.04, 1.21)	1.12 (1.06, 1.18)
	*P*=0.005	*P*<0.001
*Smoking status*		
Non-smoker/unknown	1.00	1.00
Current smoker	1.09 (1.00, 1.20)	1.14 (1.07, 1.22)
Ex-smoker	1.10 (0.99, 1.22)	1.03 (0.96, 1.10)
	*P*=0.061	*P*<0.001
*Grade*		
Well	1.00	1.00
Moderate	0.94 (0.79, 1.13)	1.06 (0.97, 1.16)
Poor/undifferentiated	1.43 (1.17, 1.75)	1.40 (1.27, 1.56)
Missing	1.15 (0.96, 1.36)	1.12 (1.01, 1.25)
	*P*<0.001	*P*<0.001
*Period of diagnosis*		
1994–96		1.00
1997–99	—	0.87 (0.82, 0.92)
2000–02		0.82 (0.76, 0.87)
		*P*<0.001

CI=confidence interval; HR=hazard ratio.

a All HRs adjusted for factors shown in relevant column (other than site), plus health board and site (colon/rectum).

b Models also stratified by receipt of chemotherapy and radiotherapy.

**Table 5:** Risk of death by adjuvant treatment^a^ for colorectal cancers diagnosed during 1994–2002: HRs with 95% CIs

	**Stage II**		**Stage III**		**Stage IV**		**Stage unknown**	
	**HR**	**95% CI**	**HR**	**95% CI**	**HR**	**95% CI**	**HR**	**95% CI**
*Chemotherapy: colorectal cancer*								
All patients^b^	0.88	(0.77, 1.01)	0.67	(0.61, 0.75)	0.83	(0.76, 0.91)	1.23	(1.03, 1.47)
Surgical patients^c^	0.96	(0.84, 1.10)	0.69	(0.62, 0.76)	0.77	(0.68, 0.88)	1.57	(1.23, 2.00)
								
*Radiotherapy: rectal cancer*								
All patients: any radiotherapy^d^^,^^e^	1.23	(0.98, 1.55)	1.06	(0.90, 1.24)	0.97	(0.83, 1.14)	1.15	(0.94, 1.40)
Surgical patients: any radiotherapy^c^^,^^d^	1.09	(0.88, 1.35)	0.88	(0.75, 1.04)	0.79	(0.62, 1.00)	1.41	(1.03, 1.94)
Surgical+radiotherapy patients: pre-operative radiotherapy^f^	1.03	(0.63, 1.66)	0.61	(0.42, 0.91)	0.70	(0.40, 1.25)	0.60	(0.36, 1.02)

CI=confidence interval; HR=hazard ratio.

a Treatment received within 1 year of diagnosis.

b Hazard ratios adjusted for age, sex, marital status, smoking status, health board, tumour extent (T), nodes (N), site, surgery receipt (yes/no), and radiotherapy (time-dependant covariate).

c Hazard ratios adjusted for age, sex, marital status, smoking status, health board, tumour extent (T), nodes (N), and site.

d Pre- or post-operative radiotherapy *vs* no radiotherapy.

e Hazard ratios adjusted for age, sex, marital status, smoking status, health board, tumour extent (T), nodes (N), surgery receipt (yes/no), and chemotherapy (time-dependant covariate).

f Comparison of pre- *vs* post-operative radiotherapy among patients treated with surgery and radiotherapy; HR adjusted for age, sex, marital status, smoking status, health board, tumour extent (T), and nodes (N).
